# Correction: The burden of chronic diseases among Australian cancer patients: Evidence from a longitudinal exploration, 2007–2017

**DOI:** 10.1371/journal.pone.0295689

**Published:** 2023-12-05

**Authors:** Rashidul Alam Mahumud, Khorshed Alam, Jeff Dunn, Jeff Gow

There is an error in the affiliations for the first author. Specifically, #3 was included in error and should not have been indicated. The correct affiliations are as follows:

Rashidul Alam Mahumud^1,2,3*^, Khorshed Alam^1,2^, Jeff Dunn^1,4,5^, Jeff Gow^1,2,6^

**1** Health Economics and Policy Research, Centre for Health, Informatics and Economic Research, University of Southern Queensland, Toowoomba, Queensland, Australia, **2** School of Commerce, University of Southern Queensland, Toowoomba, Queensland, Australia, **3** Health and Epidemiology Research, Department of Statistics, Rajshahi, Bangladesh, **4** Cancer Research Centre, Cancer Council Queensland, Fortitude Valley, Queensland, Australia, **5** Prostate Cancer Foundation of Australia, St Leonards, New South Wales, Australia, **6** School of Accounting, Economics and Finance, University of KwaZulu-Natal, Durban, South Africa

Additionally, there was an error in the corresponding author’s listed email addresses. Correspondence regarding this article should only be sent to the following email address: rashed.mahumud@usq.edu.au.
