# Supplementary material for: The burden of chronic diseases among Australian cancer patients: Evidence from a longitudinal exploration, 2007-2017
Source: PLoS One. 2020 Feb 12;15(2):e0228744. doi: 10.1371/journal.pone.0228744 (PMC7015395; doi:10.1371/journal.pone.0228744)
Supplement: Attachment — Submitted filename: Response to Reviewers.docx [file pone.0228744.s001.docx]

**Response to the editor’s and reviewer’s comments**

Date: 20 November 2019

Dear Editor and Reviwer(s),

Thank you for giving us an opportunity to revise our manuscript entitled “*The burden of chronic diseases among Australian cancer patients: Evidence from a longitudinal exploration, 2007-2017*”. We found the reviewers’ comments/feedback very helpful in improving the manuscript and we have revised the manuscript accordingly. Please find attached the revised manuscript. We have no conflict of interest to declare. The manuscript has not been published in any other journal. Our point-by-point comments on the suggested revisions are below.

Best regards,

Rashidul Alam Mahumud (corresponding author)

PhD Candidate, MPH, MSc

On behalf of all of the co-authors

Health Economics Research

Health Systems and Population Studies Division

International Centre for Diarrhoeal Disease Research, Bangladesh (icddr,b)

Dhaka 1212, Bangladesh.

Journal Requirements:

1. When submitting your revision, we need you to address these additional requirements. Please ensure that your manuscript meets PLOS ONE's style requirements, including those for file naming. The PLOS ONE style templates can be found at
   <http://www.journals.plos.org/plosone/s/file?id=wjVg/PLOSOne_formatting_sample_main_body.pdf> and <http://www.journals.plos.org/plosone/s/file?id=ba62/PLOSOne_formatting_sample_title_authors_affiliations.pdf>

**Response:** The manuscript has been revised according to the PLOS ONE style templates*.*

2. We note that you have indicated that data from this study are available upon request. PLOS only allows data to be available upon request if there are legal or ethical restrictions on sharing data publicly. For information on unacceptable data access restrictions, please see <http://journals.plos.org/plosone/s/data-availability#loc-unacceptable-data-access-restrictions>.

In your revised cover letter, please address the following prompts:

a) If there are ethical or legal restrictions on sharing a de-identified data set, please explain them in detail (e.g., data contain potentially identifying or sensitive patient information) and who has imposed them (e.g., an ethics committee). Please also provide contact information for a data access committee, ethics committee, or other institutional body to which data requests may be sent.
b) If there are no restrictions, please upload the minimal anonymized data set necessary to replicate your study findings as either Supporting Information files or to a stable, public repository and provide us with the relevant URLs, DOIs, or accession numbers. Please see <http://www.bmj.com/content/340/bmj.c181.long> for guidelines on how to de-identify and prepare clinical data for publication. For a list of acceptable repositories, please see <http://journals.plos.org/plosone/s/data-availability#loc-recommended-repositories>.

We will update your Data Availability statement on your behalf to reflect the information you provide.

**Response:** The cover letter has been revised by incorporating an ethical or legal restrictions on sharing a de-identified data set. Data access committee or ethics committee is legal authority for sharing a de-identified data set. The revised ‘data availability statement’ has been updated on the submission system.

3. Please include your tables as part of your main manuscript and remove the individual files. Please note that supplementary tables (should remain/ be uploaded) as separate "supporting information" files

**Response:** All tables have been included as part of main manuscript. Please see on the revised manuscript.

| Reviewer-I | | | |
| --- | --- | --- | --- |
| No | Reviewer’s comments | Authors’ response | References |
| 1. | Thank you for the opportunity to read and comment on your manuscript, this is an interesting study. I have some comments and suggestions, which I list below. | The authors express gratitude to the reviewer for the appreciation. We found the reviewers’ comments/feedback very helpful in improving the manuscript. The manuscript has been revised accordingly. | Please see the revised manuscript. |
| 2. | 1) The title is quite broad, and you describe the burden as “emerging”. I’m not clear if by ‘burden’ you are referring to the presence of chronic disease in cancer patients (and the complexity this comorbidity presents in the care of cancer patients), and what the evidence is from your study to suggest this is an emerging burden? | The authors would like to thank the reviewer for helpful and insightful comments. The title of the paper has been revised by deleting the word “emerging”. The revised title is: “The burden of chronic diseases among Australian cancer patients: Evidence from a longitudinal exploration, 2007-2017”. | Please see the revised title of this manuscript. |
| 3. | I would have liked more detail about the cancer patient population in this study, specifically the timing of when the cancer had been diagnosed (in relation to the timing of the survey) and the type of cancer diagnosed. The latter in particular is really important in relation to this study, given that the aetiology of cancers can vary according to their type, and that some cancers share common risk factors with some of the chronic conditions you investigated (e.g. smoking is strongly associated with both lung cancer and emphysema). | The authors fully appreciated the reviewer’s point. The target population of this study, diagnosed with cancer, were generated from the HILDA survey. The aim of this study is to examine the relationship between the health burden of cancer patients and their experiences with comorbidity conditions over time. The nature of the relationship might vary depending on cancer stages and types of cancer. However, the authors were not able to conduct cancer-specific analysis due to the paucity of data related to cancer stages and types of cancer in the HILDA survey. We have explained this issue as one of the limitations of the study which could be explored in future research. | Please see page 18 (lines 26-29) |
| 4. | In addition, was the distribution of cancer types similar across each wave of the study? | The data on study participants diagnosed with cancer were restricted to four waves (i.e., wave-7, wave-9, wave-13 and wave-17) when detailed health data was collected. We have excluded wave-3 from the analysis due to data limitations related to the outcome variable (i.e., the number of chronic comorbid conditions). The distribution of cancer types might be different across the survey waves. For example, if participants diagnosed with a particular type of cancer in wave-7, they might be exposed to other cancers in wave-8 or later waves due to advanced course of treatment, delay in diagnosis, stage of disease at diagnosis, the severity of disease and multiple exposures to comorbid conditions. | Not applicable |
| 5. | Are the changes in chronic comorbid conditions over time presented in Figure 1 a like-for-like comparison among the cancer patient populations? | Figure 1 shows that the overall trend of chronic comorbid conditions among cancer patients. The intent of Figure-1 is to investigate the magnitude of the changes in disease patterns over time. | Please see revised Figure-1. |
| 6. | 3) Explanatory variables. i) Is there a main exposure of interest? | We thank the reviewer for this insightful comment. The aim of the present study was to examine the longitudinal nature of chronic comorbid conditions among cancer patients over time. The main exposure of interest was ‘health status burden’, which was derived from the short form-36 (SF-36). We did not find any evidence in relation to the level of health status burden. In this study, we have proposed three different levels of health status burden: (i) high burden if the short form-36 (SF-36) score < 50.00, (2) moderate burden if 50.00 ≤SF-36 score < 90.00, and (3) no burden if SF-36 score ≥ 90.00. | Please see page 9 (lines 9-23). |
| 7. | ii) Why did you include the variable 'satisfaction with household members' as a predictor in your analysis, how does this relate to comorbidity in cancer patients? Was it considered to be a proxy variable for stress?! | The authors appreciate the reviewer’s concern. We have revised the analysis by dropping this predictor (i.e., satisfaction with household members). | Please see page 9 (line 7) and revised Tables. |
| 8. | A conceptual framework diagram may help to explain your assumptions of the relationships between your explanatory variables and outcome variable. | A new section has been developed by incorporating a conceptual framework. This framework explains the hypothesised relationships between the outcome variable and explanatory variables. | Please see page 6 (lines 22-30) and page 7 (lines 1-7). |
| 9. | 4) Table 1: it would be interesting to include a summary of the distribution of the number of chronic conditions according to the wave of the study. | All analyses have been updated as suggested by the reviewer. Table 1 has been revised to include a summary of the distribution of the number of chronic conditions according to the wave of the study. | Please see updated Table 1. |
| 10. | Minor comments 5) Avoid vague statements, specifically:  i) Top of page 4: "Existing research recognises the critical role played by comorbid chronic conditions among cancer patients". | We thank the reviewer for their constructive comments. The text has been revised. The sentence now reads: “A number of studies found that comorbid chronic conditions were more pronounced among cancer patients (Ng et al. 2018; Sarfati et al. 2009; Gross et al. 2007; Cuthbert et al. 2018; Sigel and Wisnivesky 2017; Sogaard et al. 2013; Lindhagen et al. 2015)”. | Please see page 5 (lines 5-7). |
| 11. | ii) Page 5: "Individuals with cancer who faced the burden of chronic comorbid conditions were investigated to see the degree of the cancer burden related to its primary evaluation as well as their ability to cope". What do you mean by the degree of the cancer burden relating to its primary evaluation? How are you quantifying ability to cope? | Authors agree with the reviewer’s concern. We have revised the text by removing the sentence. | Please see revised manuscript pages 6 and 7. |

| Reviewer-II | | | |
| --- | --- | --- | --- |
| No | Reviewer’s comments | Author’s response | References |
| 1. | The present manuscript titled ‘The emerging burden of chronic diseases among Australian cancer patients: Evidence from a longitudinal exploration, 2003-2017' presents a prospective longitudinal design using data from the HILDA Australian survey. Authors have applied a fixed-effect negative binomial regression model to predict the potential factors of occurrence in chronic comorbid conditions. In general, the theme of the manuscript is important and relevant. However, I found some key points unexplained or not covered in the present form of the manuscript. I would suggest a revision (given below) before the acceptance of the manuscript for publication in the PloS ONE. | The authors express gratitude to the reviewer for their thorough and constructive critique of our manuscript. We found all of the reviewer’s comments and suggestions very helpful in improving the manuscript. We have revised the manuscript accordingly. | Please see the revised manuscript. |
| 2. | My suggestions/comments are as follows: (1) Introduction: The Introduction provides information concerning cancer and comorbid conditions worldwide, but does not provide enough information regarding the Australian reality. I suggest the authors explain better how this study is important to the cancer epidemiology field, specifying the challenges, particularly to Australia. | The introduction section has been revised by adding information regarding the significance of the study in the context of Australia.  There are approximately one million Australians living after being diagnosed with cancer in the last couple of decades (Ng et al. 2018). However, the majority of cancer patients suffer from chronic diseases or conditions, commonly referred to as comorbidity. The risk of having comorbidity increases during treatment as well as oncology follow-up periods (AIHW 2019, 2018; WHO 2018), which adversely affects treatment choices. Chronic comorbid conditions of cancer patients contribute to a major clinical challenge in terms of cancer diagnosis, the course of treatment and disease management (Stairmand et al. 2015) and leads to an increased risk of hospitalisation, reduced quality of life, increased mortality, and increased financial burden on healthcare systems including Australia’s (Sarfati et al. 2016; Carstensen et al. 2012; Sogaard et al. 2013). It may also adversely impact an individual’s access to advance cancer treatments (e.g., chemotherapy and radiotherapy) and the effectiveness of that treatment (Pilleron et al. 2019). This is a substantial prognostic factor for the long-term survival of cancer patients. The chance of improving health status and completing a course of cancer treatment in the presence of comorbidities is significantly lower among cancer patients (Ng et al. 2018; Cuthbert et al. 2018; Sarfati et al. 2009; Gurney, Sarfati, and Stanley 2015; Elliott et al. 2004), and is associated with a higher rate of mortality depending on the severity of disease and associated comorbidity (Sogaard et al. 2013). For instance, mortality rate was substantially higher among cancer patients with comorbidities (47%) compared with cancer patients without comorbidities (34%) (Van Hemelrijck et al. 2016). Given the clinical significance of comorbidity among cancer patients and its high prevalence in cancer survivors, it is essential to have a measure for quantifying the likely effects on cancer outcomes (Pule et al. 2018). A better understanding of comorbidities among cancer patients can provide evidence as well as direction for prevention, management and treatment of chronic diseases. | Please see pages 3-6. |
| 3. | (2) Methods: Data source: I suggest the authors provide more information concerning HILDA Australian survey. In the text, the authors have described information for five waves but this information is unclear when we analyze the figures and tables. It is unclear why the authors selected five waves with cancer-related information but have just presented data for three waves in Figure 1. | The authors are thankful to the reviewer for raising the issue. The present study participants were restricted to diagnosed cancer patients. We have revised our analysis according to the reviewer's concern. Data were restricted to four waves (i.e., wave-7, wave-9, wave-13 and wave-17) based on the availability of data related to cancer diagnosis. We have excluded wave-3 in the revised analysis due to limited data related to the outcome variable (i.e., the number of chronic comorbid conditions). All tables and figures have been checked carefully and are now consistent across the survey waves. | Please see page 7 (lines 21-32). |
| 4. | Outcome variable: The authors have cited that “there is no gold-standard method to measuring comorbidity status in the context of cancer patients”, then the authors have listed some methods for measuring comorbidity status applied to the literature. As a reader, I would like to know why the authors have chosen the count of comorbid conditions? It was a single count? How this information was organized? Please, provide more information explaining these details. | We thank the reviewer for this insightful and constructive comment. The text has been revised by explaining details as we cited one review study that identified that at least 21 approaches to measuring comorbidity status (Sarfati 2012). The selection of approach depends on the research question, data availability, population studied, and the nature of study design as well as perspective. A number of methods related to measuring comorbidity status had been used in the context of cancer-related studies including exploration of the impact of single conditions (such as diabetes or congestive heart failure) (Gross et al. 2006; Meyerhardt et al. 2003; Sarfati et al. 2011), single condition counts (Tammemagi et al. 2003; Satariano and Ragland 1994; Sarfati, Gurney, Stanley, Salmond, et al. 2014), weighted indices (Klabunde et al. 2007; Sarfati, Gurney, Stanley, Lim, et al. 2014; Fleming et al. 2003; Sarfati, Gurney, Stanley, Salmond, et al. 2014; Charlson et al. 1987), and organ-based systems (Miller et al. 1992; Piccirillo 2000; Piccirillo et al. 2004). Although all these approaches aim to evaluate the same underlying construct, they vary in terms of the study purpose for which the measures were performed. These approaches were varied in the context of study perspective and design. The simplest approach to measuring comorbidity status is to investigate the distribution of individual comorbid conditions and to treat them independently and/or to combine them by summing the total number of conditions (Mandelblatt et al. 2001). In this study, a single condition counts approach was performed to measuring comorbidity status due to the paucity of cancer-related data in the HILDA survey.  Cancer patients reporting chronic condition(s) were considered an outcome variable in the analysis. Chronic comorbid conditions included being diagnosed with serious chronic illness, including arthritis or osteoporosis, heart disease, diabetes, hypertension, mental illness, or circulatory conditions. The count of chronic health conditions was measured for each respondent based on the number of disease exposures and who had been prescribed medication for their illness. If the respondents had exposed to two or more chronic comorbid conditions, it was counted two or more times responses. | Please see page 8 (lines 6-28). |

| 5. | Explanatory variables: The authors applied several demographic, socio-economic and health and lifestyle-related variables in this study. For each variable, there is a specific categorization method. For some variables the rationale of applying the scale is unclear, an example is the application of the SF-36 scale. I know what the survey means but it is necessary to explain it in the text.  I didn’t get the rationale of why the authors have chosen this quality of life scale to justify health burden levels and also how the score was made in this study. I also didn’t see any information as supplementary material of these data. Please, I suggest the authors clarify them. | We fully appreciate the reviewer’s concerns. The section of ‘explanatory variables’ has been revised now to accommodate these issues. We have added text which explains the rationale of measuring the quality of life scores as follows:  The quality of life scores was measured using the medical outcomes study short-form (SF-36) (RAND Corporation n.d.). The SF-36 is one of the most common generic measures of health-related quality of life, which is widely used to assess the burden of disease in different country settings (Yarlas et al. 2011). It expresses the psychometric properties to enable profiling of physical functional health and well-being and to quantify disease burden across eight domains including physical functioning, role-physical, body pain, general health, vitality, social functioning, role-emotional, and mental health. Considering these dimensions, the total score on each SF-36 subscale is referred to as points between 0 to 100, labelling ‘worst imaginable health’ and ‘best imaginable health state’, respectively. It is signified that higher scores represent better health status. A recent review study confirmed that several studies used a total score of SF-36 items to derive quality of life scores across eight domains of SF-36 (Lins and Carvalho 2016). The levels of health status burden were proposed in this study based on the magnitude of quality of life scores as follows: (1) high burden if the short form-36 (SF-36) score < 50.00, (2) moderate burden if 50.00 ≤SF-36 score < 90.00, and (3) no burden if SF-36 score ≥ 90.00. The level of health status burden captured the severity of disease for cancer patients. | Please see page 8 (lines 30-31), page 9 (lines 1-33). |
| --- | --- | --- | --- |
| 6. | Statistical analysis: What the authors understand as an “insignificant predictors were not included in the adjusted model”? Please, provide more information and explain it. I understand that the adjusted model was applied as a fixed-effect negative binomial regression. But what method was applied and defined as unadjusted? I suggest again the authors clarify and explain better this important information for future readers of this manuscript. | We thank the reviewer for this insightful and constructive comments. The text has been revised by deleting the sentence “the chi-square tests (or one-way analysis of variance where appropriate) to examine the association between the comorbidity status and the potential confounders of greatest interest”. In the analytical exploration, the adjusted fixed-effect negative binomial regression model was used to identify the potential factors that had a significant role in the exposure to chronic comorbid conditions. The predictor variables were included in the adjusted model only if any label of the predictor was significant at 5% or less risk level in the unadjusted model, which in turn was used to adjust for the effects of other potential confounders. We have performed unadjusted analysis using only separated explanatory variables for the following reasons: (1) primary screening of the selection of qualified predictors, which were added in the adjusted model, (2) although the chi-square tests (or one-way analysis where appropriate) are only used to find the association between outcome and explanatory variables. However, the majority of the predictor variables were categorical nature with two or more labels in this study. Therefore, we have performed an un-adjusted analysis to find the association between outcome and the labels of explanatory variables. However, insignificant predictors were not included in the adjusted model. | Please see page 10 (lines 5-22). |
| 7. | (3) Results: - The quality of the Figures is not good. Please, provide the Figures in better quality. I simply cannot read the legend of Figure 3. | All figures have been updated by ensuring better quality. | Please see the revised figures. |
| 8. | - Why the authors have only shown information for three waves in Figure 1 if in the Methods section were described five waves? The authors have applied a Cochran- Armitage trend test. It was the rationale of present the results for three waves? Please, explain it. | All analyses have been updated by ensuring the consistency of data in the figures and tables. We have used four waves for all analyses, while we have excluded wave-3 due to the paucity of data related to chronic comorbid conditions. | Pleace see revised figures. |
| 9. | - As a reader, It will be more interesting to see in Table 2 the results for each comorbid condition such as the authors have shown in Table 1 (0 chronic diseases; 1-2 chronic diseases; 3 or more chronic diseases). | The authors are thankful for the reviewer's advice. As a response to comment-4, the number of chronic comorbid condition(s) was considered an outcome variable in the analytical exploration. The count of chronic health conditions was measured for each respondent based on the number of disease exposures and who had been prescribed medication for their illness. If the respondent had been exposed to two or more comorbid conditions, it was counted two or more times responses. In the regression model, the dependent variable (number of chronic comorbid conditions) was characterised as a count measure. Based on the characteristics of data and assumptions, the fixed-effect negative binomial regression model was used to identify the potential factors that had a significant role in the exposure to chronic comorbid conditions. | Not applicable. |
| 10. | - I didn’t see any information as supplementary material of all the several variables analyzed in this study. | All analyses and data were included in the manuscript. | Please see revised tables and figures. |
| 11. | (4) Discussion: Your discussion is interesting and you made an effort to compare your results with the results of previous studies. However, what is your specific recommendations based on the results you produced to Australian reality? Please, provide more information on what has been studied in Australia and why your study is important on this topic. | We thank the reviewer for this very constructive feedback and we apologize for this lack of clarity. We have reworked this section incorporating specific recommendations based on the study findings. The study results could be used to better outline the management of a sequelae course of treatment for those who should undergo more intensive physical rehabilitation aimed at reducing the risk of adverse health outcomes. Given the clinical significance of comorbidity in cancer survivors, this study may play a significant role in providing comprehensive evidence for health care providers, including physical therapists and oncologists, who should be aware of the unique problems that challenge this population and who should advocate for prevention and evidence-based interventions. Finally, a greater awareness of the importance of managing a patients' overall health status within the context of comorbidity is warranted together with emphasized research on comorbidity to generate an appropriate scientific basis on which to build evidence-based care guidelines for patients with chronic comorbid conditions.  This study has some strengths including the use of a prospective longitudinal design of long term follow-ups and the application of well-validated and reliable longitudinal wave measures of the impacts of a cancer diagnosis on the burden of chronic comorbid conditions of individuals over the 2007-2017 period. The study population was captured in different dimensions including ethnically, geographically, and socio-economically diverse groups. Furthermore, this study included several potential confounding factors such as health status burden, the severity of the disability level as well as life satisfaction (e.g., employment, financial situation and social supports) that were not considered in previous studies. | Please see pages 13-19. |
| 12. | (4) Minor comments: Some sentences do not read well. Please consider revising. | The whole manuscript has been revised extensively. | Please see revised manuscript. |

References

Australian Institute of Health and Welfare. 2018. “Australia’s Health 2018.” Australia’s health series no. 16. AUS 221. Canberra: AIHW.

———. 2019. “Australian Burden of Disease Study: Impact and Causes of Illness and Death in Australia 2015.” Australian Burden of Disease series no. 19. Cat. no. BOD 22. Canberra: AIHW.

Carstensen, John, David Andersson, Malin Andre, Sven Engstrom, Henrik Magnusson, and Lars Axel Borgquist. 2012. “How Does Comorbidity Influence Healthcare Costs? A Population-Based Cross-Sectional Study of Depression, Back Pain and Osteoarthritis.” *BMJ Open* 2: e000809. https://doi.org/10.1136/ bmjopen-2011-000809.

Charlson, Mary E, Peter Pompel, Kathy L Ales, and C Ronald MacKenzie. 1987. “A New Method of Classifying Prognostic Comorbidity in Longitudinal Studies: Development and Validation.” *Journal of Chronic Disability* 40 (5): 373–83.

Cuthbert, Colleen A., Brenda R. Hemmelgarn, Yuan Xu, and Winson Y. Cheung. 2018. “The Effect of Comorbidities on Outcomes in Colorectal Cancer Survivors: A Population-Based Cohort Study.” *Journal of Cancer Survivorship* 12 (6): 733–43. https://doi.org/10.1007/s11764-018-0710-z.

Elliott, Barbara A., Colleen M. Renier, Irina V. Haller, and Thomas E. Elliott. 2004. “Health-Related Quality of Life (HRQoL) in Patients with Cancer and Other Concurrent Illnesses.” *Quality of Life Research* 13 (2): 457–62. https://doi.org/10.1023/B:QURE.0000018476.11278.35.

Fleming, Steven T., Kevin A. Pearce, Kathleen McDavid, and Dmitri Pavlov. 2003. “The Development and Validation of a Comorbidity Index for Prostate Cancer among Black Men.” *Journal of Clinical Epidemiology* 56 (11): 1064–75. https://doi.org/10.1016/S0895-4356(03)00213-0.

Gross, Cary P., Zhenchao Guo, Gail J. McAvay, Heather G. Allore, Mary Young, and Mary E. Tinetti. 2006. “Multimorbidity and Survival in Older Persons with Colorectal Cancer.” *Journal of the American Geriatrics Society* 54 (12): 1898–1904. https://doi.org/10.1111/j.1532-5415.2006.00973.x.

Gross, Cary P., Gail J. McAvay, Zhenchao Guo, and Mary E. Tinetti. 2007. “The Impact of Chronic Illnesses on the Use and Effectiveness of Adjuvant Chemotherapy for Colon Cancer.” *Cancer* 109 (12): 2410–19. https://doi.org/10.1002/cncr.22726.

Gurney, Jason, Diana Sarfati, and James Stanley. 2015. “The Impact of Patient Comorbidity on Cancer Stage at Diagnosis.” *British Journal of Cancer* 113 (9): 1375–80. https://doi.org/10.1038/bjc.2015.355.

Hemelrijck, Mieke Van, Yasin Folkvaljon, Jan Adolfsson, Olof Akre, Lars Holmberg, Hans Garmo, and Pär Stattin. 2016. “Causes of Death in Men with Localized Prostate Cancer: A Nationwide, Population-Based Study.” *BJU International* 117 (3): 507–14. https://doi.org/10.1111/bju.13059.

Klabunde, Carrie N., Julie M. Legler, Joan L. Warren, Laura Mae Baldwin, and Deborah Schrag. 2007. “A Refined Comorbidity Measurement Algorithm for Claims-Based Studies of Breast, Prostate, Colorectal, and Lung Cancer Patients.” *Annals of Epidemiology* 17 (8): 584–90. https://doi.org/10.1016/j.annepidem.2007.03.011.

Lindhagen, Lars, Mieke Van Hemelrijck, David Robinson, Pär Stattin, and Hans Garmo. 2015. “How to Model Temporal Changes in Comorbidity for Cancer Patients Using Prospective Cohort Data.” *BMC Medical Informatics and Decision Making* 15 (1): 1–8. https://doi.org/10.1186/s12911-015-0217-8.

Lins, Liliane, and Fernando Martins Carvalho. 2016. “SF-36 Total Score as a Single Measure of Health-Related Quality of Life: Scoping Review.” *SAGE Open Medicine* 4: 1–12. https://doi.org/10.1177/2050312116671725.

Mandelblatt, Jeanne S, Arlene S Bierman, Karen Gold, Yi Zhang, H Ng, Nancy Maserejan, Nealj Meropol, and Rebecca A Silliman. 2001. “Constructs of Burden of Illness in Older Patients with Breast Cancer: A Comparison of Measurement Methods.” *Health Services Research* 36 (6): 1085–1107. https://doi.org/10.1111/j.1475-6773.2007.00786.x.

Meyerhardt, JA, PJ Catalano, DG Haller, RJ Mayer, JS Macdonald, AB Benson, and CS Fuchs. 2003. “Impact of Diabetes Mellitus on Outcomes in Patients with Colon Cancer.” *Journal of Clinical Oncology* 21 (3): 433–40. https://doi.org/10.1200/JCO.2003.07.125.

Miller, Mark D, A Hind Rifai, Cynthia F Parrdis, Patricia R Wouck, Jacqueline A Stack, A Hindi Rifai, Benoit Mulsant, and Charless F Reynolds. 1992. “Rating Chronic Medical Illness Burden in Geropsychiatric Practice and Research: Application of the Cumulative Illness Rating Scale.” *Psychiatry Research* 41: 237–48.

Ng, Huah Shin, Bogda Koczwara, David Roder, and Agnes Vitry. 2018. “Changes in the Prevalence of Comorbidity in the Australian Population with Cancer, 2007–2014.” *Cancer Epidemiology* 54 (December 2017): 56–62. https://doi.org/10.1016/j.canep.2018.03.010.

Piccirillo, Jay, Edward Spitznagel, Nisha Vermani, Irene Costas, and Mark Schnitzler. 2004. “Comparison of Comorbidity Indices for Patients with Head and Neck Cancer.” *Medical Care* 42 (5): 482–86. https://doi.org/10.1097/01.mlr.0000124254.88292.a1.

Piccirillo, JF. 2000. “Importance of Comorbidity in Head and Neck Squamous Cell Cancer.” *Laryngoscope* 110 (April): 593–602.

Pilleron, Sophie, Diana Sarfati, Maryska Janssen-Heijnen, Jérôme Vignat, Jacques Ferlay, Freddie Bray, and Isabelle Soerjomataram. 2019. “Global Cancer Incidence in Older Adults, 2012 and 2035: A Population-Based Study.” *International Journal of Cancer* 144 (1): 49–58. https://doi.org/10.1002/ijc.31664.

Pule, Lettie, Elizabeth Buckley, Theophile Niyonsenga, David Banham, and David Roder. 2018. “Developing a Comorbidity Index for Comparing Cancer Outcomes in Aboriginal and Non-Aboriginal Australians.” *BMC Health Services Research* 18 (1): 1–8. https://doi.org/10.1186/s12913-018-3603-y.

RAND Corporation. n.d. “36-Item Short Form Survey Instrument (SF-36).” RAND Health Care Communications. Santa Monica, CA 90407-2138. Accessed November 11, 2019. https://www.rand.org/health-care/surveys_tools/mos/36-item-short-form.html.

Sarfati, Diana. 2012. “Review of Methods Used to Measure Comorbidity in Cancer Populations: No Gold Standard Exists.” *Journal of Clinical Epidemiology* 65 (9): 924–33. https://doi.org/10.1016/j.jclinepi.2012.02.017.

Sarfati, Diana, Jason Gurney, Bee Teng Lim, Nasser Bagheri, Andrew Simpson, Jonathan Koea, and Elizabeth Dennett. 2016. “Identifying Important Comorbidity among Cancer Populations Using Administrative Data: Prevalence and Impact on Survival.” *Asia-Pacific Journal of Clinical Oncology* 12: e47–56.

Sarfati, Diana, Jason Gurney, James Stanley, Bee Lim, and Claire McSherry. 2014. “Development of a Pharmacy-Based Comorbidity Index for Patients with Cancer.” *Medical Care* 52 (7): 586–93. https://doi.org/10.1097/MLR.0000000000000149.

Sarfati, Diana, Jason Gurney, James Stanley, Clare Salmond, Peter Crampton, Elizabeth Dennett, Jonathan Koea, and Neil Pearce. 2014. “Cancer-Specific Administrative Data-Based Comorbidity Indices Provided Valid Alternative to Charlson and National Cancer Institute Indices.” *Journal of Clinical Epidemiology* 67 (5): 586–95. https://doi.org/10.1016/j.jclinepi.2013.11.012.

Sarfati, Diana, Sarah Hill, Tony Blakely, Bridget Robson, Gordon Purdie, Elizabeth Dennett, Donna Cormack, and Kevin Dew. 2009. “The Effect of Comorbidity on the Use of Adjuvant Chemotherapy and Survival from Colon Cancer: A Retrospective Cohort Study.” *BMC Cancer* 9 (116): 1–10. https://doi.org/10.1186/1471-2407-9-116.

Sarfati, Diana, Lavinia Tan, Tony Blakely, and Neil Pearce. 2011. “Comorbidity among Patients with Colon Cancer in New Zealand.” *The New Zealand Medical Journal* 124 (1338): 76–88.

Satariano, William A., and David R. Ragland. 1994. “The Effect of Comorbidity on 3-Year Survival of Women with Primary Breast Cancer.” *Annals of Internal Medicine* 120 (2): 104–10. https://doi.org/10.7326/0003-4819-120-2-199401150-00002.

Sigel, Keith, and Juan P. Wisnivesky. 2017. “Comorbidity Profiles of Patients with Lung Cancer: A New Approach to Risk Stratification?” *Annals of the American Thoracic Society* 14 (10): 1512–13. https://doi.org/10.1513/annalsats.201706-442ed.

Sogaard, Mette, Reimar Wernich Thomsen, Kristine Skovgaard Bossen, Henrik Toft Sørensen, and Mette Nørgaard. 2013. “The Impact of Comorbidity on Cancer Survival: A Review.” *Clinical Epidemiology* 5 (Suppl 1): 3. https://doi.org/10.2147/clep.s47150.

Stairmand, J., Louise Signal, D. Sarfati, C. Jackson, L. Batten, M. Holdaway, and C. Cunningham. 2015. “Consideration of Comorbidity in Treatment Decision Making in Multidisciplinary Cancer Team Meetings: A Systematic Review.” *Annals of Oncology* 26 (7): 1325–32. https://doi.org/10.1093/annonc/mdv025.

Tammemagi, C. Martin, Christine Neslund-Dudas, Michael Simoff, and Paul Kvale. 2003. “Impact of Comorbidity on Lung Cancer Survival.” *International Journal of Cancer* 103 (6): 792–802. https://doi.org/10.1002/ijc.10882.

World Health Organization (WHO). 2018. “Noncommunicable Diseases.” Key Facts. 2018. https://www.who.int/en/news-room/fact-sheets/detail/noncommunicable-diseases.

Yarlas, Aaron S., Michelle K. White, Min Yang, Renee N. Saris-Baglama, Peter Galthen Bech, and Torsten Christensen. 2011. “Measuring the Health Status Burden in Hemodialysis Patients Using the SF-36® Health Survey.” *Quality of Life Research* 20 (3): 383–89. https://doi.org/10.1007/s11136-010-9764-8.
