# Supplementary material for: The burden of chronic diseases among Australian cancer patients: Evidence from a longitudinal exploration, 2007-2017
Source: PLoS One. 2020 Feb 12;15(2):e0228744. doi: 10.1371/journal.pone.0228744 (PMC7015395; doi:10.1371/journal.pone.0228744)
Supplement: Attachment — Submitted filename: Response to Reviewers.docx [file pone.0228744.s002.docx]

**Response to the editor’s and reviewer’s comments**

Date: 22 January 2020

Dear Editor and Reviwer(s),

Thank you for giving us an opportunity to revise our manuscript entitled “*The burden of chronic diseases among Australian cancer patients: Evidence from a longitudinal exploration, 2007-2017*”. We found the reviewers’ comments/feedback very helpful in improving the manuscript and we have revised the manuscript accordingly. Please find attached the revised manuscript. We have no conflict of interest to declare. The manuscript has not been published in any other journal. Our point-by-point comments on the suggested revisions are below.

Best regards,

Rashidul Alam Mahumud (corresponding author)

PhD Candidate, MPH, MSc

On behalf of all of the co-authors

Health Economics Research

Health Systems and Population Studies Division

International Centre for Diarrhoeal Disease Research, Bangladesh (icddr,b)

Dhaka 1212, Bangladesh.

| Reviewer-I | | | |
| --- | --- | --- | --- |
| No | Reviewer’s comments | Authors’ response | References |
| 1. | Thank you for the opportunity to read the revised version of your manuscript. I have a couple of further comments: | The authors express gratitude to the reviewer for the appreciation. We found the reviewers’ comments/feedback very helpful in improving the manuscript. The manuscript has been revised accordingly. | Please see the revised manuscript. |
| 2. | (Using your numbering system of the original comments - comment 3: (page 18, lines 26-29) you have used the word ‘emerging’ in this sentence, please clarify what this means. For example, are you saying that the prevalence of comorbidity among cancer patients has increased over time? I think you need to be more explicit. | The authors would like to thank the reviewer for helpful and insightful comments. The word ‘emerging’ has been delected from the revised manuscript. | Please see page 16 (lines 32-33). |
| 3. | Likewise the sentence “The authors were not able to estimate the cancer-specific analysis due to the paucity of relevant data” – what do you mean by cancer-specific? According to cancer type? | The authors fully appreciated the reviewer’s point. The sentence has been revised ‘The authors were not able to estimate the cancer type analysis due to the paucity of relevant data’. | Please see page 17 (lines 1-2) |

| Reviewer-II | | | |
| --- | --- | --- | --- |
| No | Reviewer’s comments | Author’s response | References |
| 1. | This is the second version of the manuscript. Authors have done considerable changes explaining in detail point-by-point according to peer reviewer's suggestions/comments and it is commendable. The revision certainly improves the quality and scope of the present manuscript. I would recommend it for possible publication in PlosOne. | The authors express gratitude to the reviewer’s comments. We found all of the reviewer’s comments and suggestions very helpful in improving the manuscript. | Not applicable |
